# Supplementary material for: Sexual violence victimisation and response among university students in sub-Saharan Africa: a scoping review protocol
Source: BMJ Open. 2024 Jan 17;14(1):e076015. doi: 10.1136/bmjopen-2023-076015 (PMC10806683; doi:10.1136/bmjopen-2023-076015)
Supplement: Supplementary data [file bmjopen-2023-076015supp001.pdf]

Electronic search strategy

Our initial search involved two databases (MEDLINE and CINAHL) using the title of this review. We then analyze the text words contained in the title and abstract of retrieved papers, and of the index, terms used to describe the articles. Here we present **full electronic initial search strategy** for MEDLINE and CINAHL database.

Full electronic search strategy for MEDLINE database.

**Database [platform]**	**Search terms**	**Limiters/Expanders**
Medline [EBSCO]	(((MM "Sex Offenses+") OR (MH "Violence+") OR (MH "Sexual Trauma") OR (MM "Sexual Harassment") OR "sexual violence" OR (MH "Gender-Based Violence") OR "sexual abuse" OR "sexual assault" OR rape)) AND (((MM "Universities") OR (MM "Students") OR "university students" OR (MH "Students, Dental") OR (MH "Students, Public Health") OR (MH "Students, Health Occupations") OR (students in higher education) OR undergraduate OR campus))	Expanders - Apply related words; Apply equivalent subjects<br>Narrow by SubjectGeographic: - africa<br>Narrow by SubjectAge: - adult: 19-44 years<br>Narrow by Language: - english<br>Search modes - Find all my search terms
CINHAL [EBSCO]	(((MM "Sex Offenses+") OR (MH "Violence+") OR (MH "Sexual Trauma") OR (MM "Sexual Harassment") OR "sexual violence" OR (MH "Gender-Based Violence") OR "sexual abuse" OR "sexual assault" OR rape)) AND (((MM "Universities") OR (MM "Students") OR "university students" OR (MH "Students, Dental") OR (MH "Students, Public Health") OR (MH "Students, Health Occupations") OR (students in higher education) OR undergraduate OR campus))	Expanders - Apply related words; Apply equivalent subjects<br>Narrow by SubjectGeographic: - africa<br>Narrow by SubjectAge: - adult: 19-44 years<br>Narrow by Language: - english<br>Search modes - Find all my search terms
ACADEMIC SEARCH PREMIER	(((MM "Sex Offenses+") OR (MH "Violence+") OR (MH "Sexual Trauma") OR (MM "Sexual Harassment") OR "sexual violence" OR (MH "Gender-Based Violence") OR "sexual abuse" OR "sexual assault" OR rape)) AND (((MM "Universities") OR (MM "Students")	Expanders - Apply related words; Apply equivalent subjects<br>Search modes - Find all my search terms<br>Narrow by Language: - english<br>Search modes - Find all my search terms

	OR "university students" OR (MH "Students, Dental") OR (MH "Students, Public Health") OR (MH "Students, Health Occupations") OR (students in higher education) OR undergraduate OR campus))	Expanders - Apply related words; Apply equivalent subjects<br>Narrow by SubjectGeographic: - africa
EMBASE [EBSCO]	#1. 'gender violence' AND 'higher learning institution'<br>#2. ('gender violence' OR (('gender'/exp OR gender) AND ('violence'/exp OR violence))) AND ('higher learning institution' OR (higher AND ('learning'/exp OR learning) AND institution))<br>#3. (sexual AND ('violence'/exp OR violence) OR 'sexual harassment'/exp OR 'sexual harassment' OR (('sex'/exp OR sex) AND offence) OR (('gender'/exp OR gender) AND based AND ('violence'/exp OR violence)) OR (sexual AND ('abuse'/exp OR abuse)) OR (sexual AND ('assault'/exp OR assault)) OR 'rape'/exp OR rape) AND (university:ti AND students:ti OR undergraduate:ti OR (college:ti AND student:ti) OR (students:ti AND in:ti AND higher:ti AND education:ti))	#4. #3 AND 'human'/de<br>#5. #4 AND [adult]/lim
PubMed [EBSCO]	(("sexual violence"[Title] OR "sexual harassment"[Title] OR "sex offence*"[Title] OR "gender based violence"[Title] OR "sexual abuse"[Title] OR "sexual assault"[Title] OR "rape"[Title]) AND ("university student*"[Title] OR "undergraduate*"[Title] OR "college student*"[Title] OR ("student*"[All Fields] AND "in higher education"[Title]))) AND ((humans[Filter]) AND (english[Filter]) AND (adult[Filter]))	Humans, English, Adult: 19-44 years
Google	What are African governments doing about sexual violence on campus?	Websites/examples:<br>https://www.globalcitizen.org/es/content/south-africa-university-gender-violence-pandor/

		https://www.un.org/africarenewal/magazine/august-november-2018/confronting-sexual-violence-schools<br>https://aau.org/ (Association of African Universities)<br>https://essa-africa.org/ (Education Sub Saharan Africa)
