# Supplementary material for: Sexual violence victimisation and response among university students in sub-Saharan Africa: a scoping review protocol
Source: BMJ Open. 2024 Jan 17;14(1):e076015. doi: 10.1136/bmjopen-2023-076015 (PMC10806683; doi:10.1136/bmjopen-2023-076015)
Supplement: Author's manuscript [file bmjopen-2023-076015.draft_revisions.pdf]

BMJ Open

BMJ Open is committed to open peer review. As part of this commitment we make the peer review history of every article we publish publicly available.

When an article is published we post the peer reviewers' comments and the authors' responses online. We also post the versions of the paper that were used during peer review. These are the versions that the peer review comments apply to.

The versions of the paper that follow are the versions that were submitted during the peer review process. They are not the versions of record or the final published versions. They should not be cited or distributed as the published version of this manuscript.

BMJ Open is an open access journal and the full, final, typeset and author-corrected version of record of the manuscript is available on our site with no access controls, subscription charges or pay-per-view fees (http://bmjopen.bmj.com).

If you have any questions on BMJ Open's open peer review process please email info.bmjopen@bmj.com

BMJ Open

Sexual violence victimization and response among university students in sub-Saharan Africa: A scoping review protocol

Journal:	*BMJ Open*
Manuscript ID	bmjopen-2023-076015
Article Type:	Protocol
Date Submitted by the Author:	31-May-2023
Complete List of Authors:	Mzilangwe, Ester; Umea Universitet, Department of Epidemiology and Global Health; Muhimbili University of Health and Allied Sciences, Psychiatry and Mental Health<br>Chande Mallya, Rehema; Muhimbili University of Health and Allied Sciences, Directorate of Library Services; Muhimbili University of Health and Allied Sciences, Directorate of Library Services<br>Kaaya, Sylvia; Muhimbili University of Health and Allied Sciences, Department of Psychiatry and Mental Health<br>Nkulu Kalengayi, Faustine Kyungu; Umea Universitet, Epidemiology and Global Health; Umea university
Keywords:	Health Services Accessibility, Health Equity, PUBLIC HEALTH, Health policy < HEALTH SERVICES ADMINISTRATION & MANAGEMENT, Organisation of health services < HEALTH SERVICES ADMINISTRATION & MANAGEMENT, Risk Factors

SCHOLARONE™ Manuscripts

Sexual violence victimization and response among university students in sub-Saharan Africa: A scoping review protocol

Ester Steven Mzilangwe[1,2], Rehema Chande[3], Sylvia Kaaya[2], Faustine Kyungu Nkulu Kalengayi[1]

1. Department of Epidemiology and Global Health, Umea Universitet, Sweden
2. Department of Psychiatry and Mental Health, Muhimbili University of Health and Allied Sciences, Tanzania
3. Directorate of Library Services, Muhimbili University of Health and Allied Sciences, Tanzania

Corresponding Author: Ester Steven Mzilangwe,
Email: ester.mzilangwe@umu.se, ester.steven@muhas.ac.tz
Telephone number: +255788202020
Department of Epidemiology and Global Health,
Address: Umeå Universitet, SE-901 87 Umeå, Sweden

For peer review only

For peer review only - http://bmjopen.bmj.com/site/about/guidelines.xhtml

ABSTRACT

Introduction: Sexual violence (SV) is highly prevalent among university campuses across the globe, resulting in devastating educational and health consequences. While several studies have been published highlighting different aspects of SV on campuses, there is no retrieved evidence from the JBI Database of Systematic Reviews and Implementation Reports, Cochrane Library, or MEDLINE via the Ovid website that has been conducted to examine comprehensive existing literature on prevalence, risk factors, victims and perpetrators, policies, laws, and actions taken by universities in responding to SV in Sub-Saharan Africa (SSA). This review aims to map the existing literature on SV victimization among university students in SSA, related response strategies, and identify gaps in the evidence.

Methods and Analysis: This review will follow Joanna Briggs Institute guidelines and will be conducted from 1st July to 30th November 2023. A team of 4 reviewers will screen eligible documents and articles for relevance from various data sources including electronic databases such as MEDLINE, EMBASE, PsychINFO, CINAHL, Google Scholar, PubMed, and websites for government and agencies. Standard information for each study will be collected and a common analytical framework for all the primary documents will be conducted.

Ethics and dissemination: This is a protocol for a scoping review, no ethical approval is required. The results will be disseminated in peer-reviewed journals, seminars, and conferences.

Registration: This review has been registered with the Open Science Framework.

Keywords: Sexual violence, sexual offenses, response services, university students, Sub-Saharan Africa

Word count: 2,278

Data statement: There is no data set available, all data that will be generated will be made available upon request.

Strengths and limitations of this study

- The review approach will cover information from broad sources, therefore, providing broader synthetic information on SV victimization and response mechanisms on university campuses in SSA
- Studies and documents that will be included in this review are conducted and prepared by other people and therefore may include the risk of bias.
- The review will be limited to articles and documents published in the English language only and therefore will lack evidence from documents and articles published in other languages.

For peer review only - http://bmjopen.bmj.com/site/about/guidelines.xhtml

INTRODUCTION

Sexual violence (SV) is a serious global public health problem and a violation of sexual rights affecting millions worldwide. [1] SV is defined as any sexual act, attempt to obtain a sexual act, unwanted sexual comments or advances, or acts to traffic, or otherwise directed, against a person's sexuality using coercion, by any person regardless of their relationship to the victim, in any setting, including but not limited to home and work. [2] Thus, sexual consent, which is defined as consent to engage in sexual activity is a prerequisite for any sexual act. [3, 4] Most victims of SV are women, and most perpetrators are men, but men and boys can also be subjected to SV. SV is commonly reported on university campuses around the world with alarmingly high estimates. [5] Globally, the prevalence rate of sexual assault on undergraduates has been estimated at 10.3% in women and 3.1% in men. [6] A national survey conducted in 2018 that involved 153 institutions in the United Kingdom with a sample of 4,491 recent graduate students found that almost two-thirds (62%) of the graduates or students had experienced SV. Among them, 50% experienced sexual harassment, and 42% sexual assault. The proportions of victimization were 70% among females, 26% among men, 61% among non-binary, and 73% among students with disability. [7] Studies from SSA indicate that the prevalence of various forms of SV and sexual harassment experienced by university students in five countries vary within and across countries from 14.3% to 78.2%.[8-12]

There are several risk factors for SV victimization in university settings. Power hierarchies can exist between teachers and students or between younger and older students. [4] Young women may have sex with a teacher or someone else they are dependent on because they need to pass a course to get financial support, in that way, promotes their subordination and dependency on men. [13, 14] Other individual risk factors for SV victimization include young age, alcohol and drug consumption, year of study, and living out of campus. [15] Other factors include previous SV victimization, multiple sex partners, and losing a parent before age two. [8, 11, 12, 15, 16] Weak monitoring systems, lack of guidelines and unprofessional administration, and community attitudes are some of the institutional and community-level factors that are associated with SV victimization. [15] At the policy level, lack of or inadequate laws and policies to address and prevent different forms of SV and protect students.

Several initiatives are implemented to address SV in universities; these include policies and guidelines to prevent and respond to SV, accessible and trauma-informed services for supporting student disclosure, reporting and complaints, and counseling and advocacy. [17] Most interventions have been developed and implemented in high-income countries. [18] Despite existing policies, there have been challenges reported in translating these policies into actions/practices in many parts of the world. A review of studies of campus policies from high-income countries and practices listed inconsistency in the definitions of what constitutes SV or sexual harassment, the unintended consequences of mandatory reporting that may include secondary victimization and stigma, the lack of inclusiveness in the development of policies, procedures, programs, and the impact of the campus climate on policy and practice as some of the gaps existing in universities. [18] Studies on students' knowledge about resources/services have shown inconsistent results; some suggest that students were knowledgeable about available services/resources whereas others indicated a lack of knowledge about available resources on the campus. [5] A study from the USA found that less than half of the students who participated reported knowing the location of the on-campus sexual assault center and most of them lacked specific knowledge on how to use them. [19] Evidence also indicates that gender influences students' knowledge about available campus SV services/resources. [19, 20] However, there are variations in the role of gender, the findings from one study from the USA indicated that men were less likely to be knowledgeable of campus SV resources/services [19] while another study found no gender differences. [20] The discrepancy was believed to be a result of variation in outreach activities of the services and the

For peer review only - http://bmjopen.bmj.com/site/about/guidelines.xhtml

ability to equally target men and women. Evidence also shows that students who had experienced unwanted sexual intercourse during the academic year were more likely to be knowledgeable of a particular campus SV resource/service (e.g. campus sexual assault center) than those without a victimization history. [19] With regards to the utilization of available campus services/resources; some qualitative studies from high-income countries suggested that some students expressed they would be comfortable using campus resources/services including personnel, such as residence assistants, professors, and staff in the wellness center, as well as campus resources and services, such as call/security boxes, (security alarm systems) security officers/escorts and transportation services. [5, 19-22]

So far, most studies that report SV in university campuses have been conducted in high-income countries and only a few in SSA countries. The latter reported high and various rates of SV victimization on campuses and related responses. However, the available evidence has not been synthesized to provide an overall picture and a clear understanding of not only the extent of the problem but also knowledge of potential victims, and perpetrators and what strategies/interventions have been implemented to respond to SV in SSA universities. Moreover, at the time of writing this protocol, there was no retrieved evidence of any existing scoping or systematic review conducted in the area of SV victimization and response among university students across Sub Sahara African countries from the JBI Database of Systematic Reviews and Implementation Reports, Cochrane Library or MEDLINE via Ovid website. In response to this gap, this scoping review will conduct a mapping of available evidence on SV victimization on campuses and different strategies used to respond to/address it in SSA universities. In addition, this scoping review will provide preliminary information to guide our research agenda.

Aim, objectives, and research questions

The overall aim of this scoping review is to map existing literature on SV victimization among university students in SSA and related response strategies and to identify gaps in the evidence. This review will provide an evidence basis for specific recommendations in future work and clearly, state gaps in the literature needed to guide the research agenda.

The specific objectives of this scoping review are

- Map existing literature on SV prevalence, types, risk factors, and common victims and perpetrators of SV among university students on campuses in SSA.
- Describe different laws, policies, strategies, and actions taken to address and prevent SV on campuses and
- identify evidence gaps.

The main questions guiding this scoping are:

1. What does existing literature tell us about SV among university students in SSA and related responses?
2. What is the gap in knowledge of the existing literature on SV victimization and response among university students in SSA?

For peer review only - http://bmjopen.bmj.com/site/about/guidelines.xhtml

METHODS

The review has been registered with the Open Science Framework and will be conducted from 1st July 2023 to 30 November 2023. The review will follow Joanna Briggs Institute (JBI) guidelines for scoping reviews. [23] These guidelines were developed from the framework proposed by Arksey and O'Malley's[24] and further enhanced by Levac, Colquhoun, and O'Brien who provided more details of what should be done in each stage of the review process. [25] The review will be reported in agreement with the JBI Manual, the Preferred Reporting Items for Systematic Reviews, and Meta-analyses extension for scoping review (PRISMA-ScR) checklist. [26] With regards to this, the steps towards conducting this review will include 1) search strategy, 2) study selection, 3) charting the data, and 4) collating, summarizing, and reporting the results. The details of what will be done in each step are explained below.

Information sources and search strategy

The reviewers will utilize the JBI-proposed three-step search strategy to identify relevant articles for this study. [23] The reviewers will first start by searching from two databases (MEDLINE and CINAHL) using the title of this review. The reviewers will then analyze the text words contained in the title and abstract of retrieved papers, and of the index, terms used to describe the articles. The second step will focus on all index terms and identified keywords from the titles and abstracts of the retrieved papers. These index terms will then be used to search all selected information sources including electronic databases such as MEDLINE, EMBASE, PsychINFO, CINAHL, Google Scholar, and PubMed. Additional sources, search terms, and keywords may be discovered and incorporated into the search strategy. The reviewers will also search for other sources and other documents including government policies and guidelines from government websites and local and international agencies that will be identified during the search process. In the third step, reviewers will use the reference list of all selected articles to search for additional studies and include them in the list. The list of all identified studies and documents will be uploaded to the EndNote reference manager software version 20.5 (clarivate), and duplicates will be removed. To minimize reporting bias, this scoping review will be conducted by two independent primary reviewers and two secondary reviewers. Reviewers will contact authors of primary studies, reviews, and policy documents for further information when additional information is required.

Eligibility criteria

As proposed by the JBI guidelines, [23] we used the population, concepts, and context (PCC) approach to formulate our inclusion criteria for the studies that will be reviewed.

Inclusion criteria

Population: The target population for this review will be the human subjects who are university students, both males and females, aged 18 years and above.

Concept: Our core concept for this review is SV prevalence, types, risk factors, common victims and perpetrators, laws, policies, strategies, and actions taken to address SV on campuses.

Context: This review will be limited to studies conducted in universities in SSA and policy documents addressing sexual violence in Universities in SSA, from the inception until 30th November 2023.

Exclusion criteria: Studies that focus on areas other than universities, the population aged less than 18 years, not focusing on the core concepts as listed above and those published in another language other than English will be excluded.

For peer review only - http://bmjopen.bmj.com/site/about/guidelines.xhtml

Study selection

Reviewers will assess the retrieved articles and grey literature/policy documents for relevance using the inclusion criteria. This will be done in two steps, first by screening the title and abstracts/summaries and then by full text whereby copies of full articles will be retrieved. A list of those studies and documents that will meet the inclusion criteria will be selected. At this stage, reviewers will convene and jointly discuss any discrepancies between the two lists. Secondary reviewers will be invited and consulted to resolve any discrepancies before moving to the next stage. The reviewers will then read the full article for assessment against the inclusion criteria for the review. The detailed flow chart of all studies/documents that were included and excluded, with clear reasons for exclusion will be developed and included in our report. Any disagreements that arise between the reviewers at this stage of the selection process will be resolved through discussion with secondary reviewers.

Charting the data

Reviewers will start by reading the primary research articles and policy documents and collecting standard information about each article. Reviewers will then extract information and enter it in the prepared data charting form using the database program Excel. Information that will be initially entered in the data charting form will include the author(s), year of publication, study location (country), study population, aims of the study, methodology, data collection tools, outcome measures, and key findings, key policy, laws, and strategies. Thereafter, the reviewers will make a broader narrative review to analyze information based on the context of the study findings and their conclusion. The charts will contain both numerical summaries and qualitative thematic analysis findings.

Collating, summarizing, and reporting the results

In this stage, reviewers will focus on identifying the implications of the study findings for policy, practice, or research. All articles included in the review will be screened for results. We will develop a thematic framework that will enable us to present a narrative account of all extracted information from the articles based on overlaps and diversities of the findings to answer our research questions. Depending on the findings from the studies we will select the best way to organize and summarize that data, these can include figures, tables, and evidence gaps maps, in a diagrammatic or tabular form, and/or in a descriptive format that aligns with our objectives and elements of the PCC as mentioned above. The decision of which method of reporting to be used will be done by involving all reviewers in the team. The outcome of this review is expected to include findings on SV victimization among university students in SSA countries and related information that will be mapped in our findings like characteristics of victims and perpetrators. Another outcome will be response systems following SV victimization and the characteristics of the response system, the structures and users, and the strength and gaps. The policy documents and guidelines will also be reviewed, and details of implementations that have been done, the strengths, as well as the gaps, will be summarized in this review.

ETHICS AND DISSEMINATION

We anticipate that these results will be disseminated through publication in a peer-reviewed journal and conferences targeting researchers, programmers, policymakers, responders, and university students across the continent. We expect that the findings of this scoping review will provide important information on SV prevalence, types, risk factors, common victims and perpetrators, laws, policies, strategies, and actions taken to address SV on campuses in SSA that will guide future work. The

For peer review only

findings will further delimit the gaps in the published data to guide the forthcoming research agenda in SSA, particularly in Tanzania.

ACKNOWLEDGMENTS

Funding statement

This work was supported by the Erling Persson's Foundation (EP) through the Department of Epidemiology and Global Health (EpiGH), Umeå University. The funding support training and supervision for the Ph.D. of the corresponding author.

The authors declare no competing interest.

Author contributions

ESM conceptualized the review approach and wrote the first draft of the manuscript. RCM reviewed and provided inputs in the methods section. FNKN identified the framework and provided general guidance in all sections. SK reviewed the framework and provided inputs on the scope of the review and methods. All authors read and approved the final manuscript.

Authors' information

ESM is a doctoral candidate at the Department of Epidemiology and Global Health, Umea University, SWEDEN, and a Psychiatrist at the Department of Psychiatry and Mental Health, Muhimbili University of Health and Allied Sciences
Email: ester.mzilangwe@umu.se, ester.steven@muhas.ac.tz
Address: Umeå Universitet, SE-901 87 Umeå, Sweden

RCM is a Senior Librarian at the Directorate of Library Services, Muhimbili University of Health and Allied Sciences
Email: rchandemallya@muhas.ac.tz, rchandemallya@gmail.com

SK is a Professor of Psychiatry at the Department of Psychiatry and Mental Health, Muhimbili University of Health and Allied Sciences
Email: skaaya@gmail.com

FNKN is a Senior Research Associate at the Department of Epidemiology and Global Health, Umeå Universitet, SWEDEN
Email: faustine.nkulu@umu.se

For peer review only - http://bmjopen.bmj.com/site/about/guidelines.xhtml

REFERENCES

1. Garcia-Moreno C PC, Devries K. . Global and regional estimates of violence against women: prevalence and health effects of intimate partner violence and non-partner sexual violence. Geneva: World Health Organization; . 2013.
2. Krug EG, Mercy JA, Dahlberg LL, Zwi AB. The world report on violence and health. The Lancet. 2002;360(9339):1083-8.
3. Beres MA. 'Spontaneous' Sexual Consent: An Analysis of Sexual Consent Literature. Feminism & Psychology. 2016;17(1):93-108.
4. Jozkowski KN, Peterson ZD. College students and sexual consent: unique insights. J Sex Res. 2013;50(6):517-23.
5. Halstead V, Williams JR, Gonzalez-Guarda R. Sexual violence in the college population: a systematic review of disclosure and campus resources and services. J Clin Nurs. 2017;26(15-16):2137-53.
6. Dills J FD, Payne G. Sexual Violence on Campus: Strategies for Prevention. . Atlanta, GA: National Center for Injury Prevention and Control, Centers for Disease Control and Prevention. 2016.
7. Report Sexual Violence at University Revolt Sexual Assault The Student Room March 2018.
8. Mezie-Okoye M-MI, Alamina F. Sexual violence among female undergraduates in a tertiary institution in Port Harcourt: prevalence, pattern, determinants and health consequences. African journal of reproductive health. 2014;18(4):79-85.
9. Ogunbode OO, Bello FA, Ogunbode AM. Sexual violence against female undergraduates in a Nigerian tertiary institution. Tropical Journal of Obstetrics and Gynaecology. 2014;31(1):99-109.
10. Agardh A, Cantor-Graae E, Ostergren PO. Youth, sexual risk-taking behavior, and mental health: a study of university students in Uganda. Int J Behav Med. 2012;19(2):208-16.
11. Fielding-Miller R, Shabalala F, Masuku S, Raj A. Epidemiology of campus sexual assault among university women in Eswatini. Journal of interpersonal violence. 2019:0886260519888208.
12. Norman ID, Aikins M, Binka FN. Sexual harassment in public medical schools in Ghana. Ghana Medical Journal. 2013;47(3):128-36.
13. Kaufman CE, Stavrou SE. 'Bus fare please': the economics of sex and gifts among young people in urban South Africa. Culture, Health & Sexuality. 2004;6(5):377-91.
14. Zembe YZ, Townsend L, Thorson A, Ekström AM. "Money talks, bullshit walks" interrogating notions of consumption and survival sex among young women engaging in transactional sex in post-apartheid South Africa: a qualitative enquiry. Globalization and Health. 2013;9(1):28.
15. Dranzoa C. Sexual Harassment at African Higher Education Institutions. International Higher Education. 2018;94:4.
16. Temesgan WZ, Endale ZM, Aynalem GL. Sexual violence and associated factors among female night college students after joining the college in Debre Markos town, North West Ethiopia, 2019. Clinical Epidemiology and Global Health. 2021;10.
17. Tertiary Education Quality and Standards Agency, Good Practice Note: Preventing and responding to sexual assault and sexual harassment in the Australian higher education sector. July 2020.
18. Warner WPJ. Sexual Violence Response and Prevention Studies of Campus Policies and Practices. Journal of School Violence. 2017.

For peer review only - http://bmjopen.bmj.com/site/about/guidelines.xhtml

19. Walsh WA, Banyard VL, Moynihan MM, Ward S, Cohn ES. Disclosure and service use on a college campus after an unwanted sexual experience. J Trauma Dissociation. 2010;11(2):134-51.
20. Hayes-Smith RM, Levett LM. Student Perceptions of Sexual Assault Resources and Prevalence of Rape Myth Attitudes. Feminist Criminology. 2010;5(4):335-54.
21. Walsh K, Danielson CK, McCauley JL, Saunders BE, Kilpatrick DG, Resnick HS. National prevalence of posttraumatic stress disorder among sexually revictimized adolescent, college, and adult household-residing women. Arch Gen Psychiatry. 2012;69(9):935-42.
22. Tsui EK, Santamaria EK. Intimate Partner Violence Risk among Undergraduate Women from an Urban Commuter College: the Role of Navigating Off- and On-Campus Social Environments. J Urban Health. 2015;92(3):513-26.
23. Institute JB. The Johanna Briggs Institute reviewers_Manual 2015.pdf2015.
24. Arksey H, O'Malley L. Scoping studies: towards a methodological framework. International Journal of Social Research Methodology. 2005;8(1):19-32.
25. Levac D, Colquhoun H, O'Brien KK. Scoping studies: advancing the methodology. Implementation science. 2010;5:1-9.
26. Tricco AC, Lillie E, Zarin W, O'Brien KK, Colquhoun H, Levac D, et al. PRISMA extension for scoping reviews (PRISMA-ScR): checklist and explanation. Annals of internal medicine. 2018;169(7):467-73.

For peer review only

For peer review only - http://bmjopen.bmj.com/site/about/guidelines.xhtml

BMJ Open

Sexual violence victimization and response among university students in sub-Saharan Africa: A scoping review protocol

Journal:	*BMJ Open*
Manuscript ID	bmjopen-2023-076015.R1
Article Type:	Protocol
Date Submitted by the Author:	23-Nov-2023
Complete List of Authors:	Mzilangwe, Ester; Umea Universitet, Department of Epidemiology and Global Health; Muhimbili University of Health and Allied Sciences, Psychiatry and Mental Health<br>Chande Mallya, Rehema; Muhimbili University of Health and Allied Sciences, Directorate of Library Services<br>Lindkvist, Marie; Umea Universitet, Department of Epidemiology and Global Health<br>Kaaya, Sylvia; Muhimbili University of Health and Allied Sciences, Department of Psychiatry and Mental Health<br>Nkulu Kalengayi, Faustine Kyungu; Umea Universitet, Epidemiology and Global Health
**Primary Subject Heading**:	Public health
Secondary Subject Heading:	Health policy, Sexual health
Keywords:	Health Services Accessibility, Health Equity, PUBLIC HEALTH, Health policy < HEALTH SERVICES ADMINISTRATION & MANAGEMENT, Organisation of health services < HEALTH SERVICES ADMINISTRATION & MANAGEMENT, Risk Factors

SCHOLARONE™
Manuscripts

Sexual violence victimization and response among university students in sub-Saharan Africa: A scoping review protocol

Ester Steven Mzilangwe[1,2], Rehema Chande[1], Marie Lindkvist[2], Sylvia Kaaya[1], Faustine Kyungu Nkulu Kalengayi[2]

1. Department of Psychiatry and Mental Health and Directorate of Library Services, Muhimbili University of Health and Allied Sciences, Tanzania
2. Department of Epidemiology and Global Health, Umea University, Sweden

Corresponding Author: Ester Steven Mzilangwe,
Email: ester.mzilangwe@umu.se, ester.steven@muhas.ac.tz
Telephone number: +255788202020
Address: 9 United Nations Road; Upanga West; P.O. Box 65001, Dar es Salaam, TANZANIA.

Data statement: The dataset of this review will be made available at the Umea University and the Muhimbili University of Health and Allied Science repositories http://dspace.muhas.ac.tz:8080/xmlui/

ABSTRACT

Introduction: Sexual violence (SV) is highly prevalent among university campuses across the globe, resulting in devastating educational and health consequences. While several studies have been published highlighting different aspects of SV on campuses, there is no retrieved review of evidence or evidence synthesis from the Joanna Briggs Institute (JBI) Database of Systematic Reviews and Implementation Reports, Cochrane Library, or MEDLINE via the Ovid website that has been conducted to examine comprehensive existing literature on prevalence, risk factors, victims and perpetrators, policies, laws, and actions taken by universities in responding to SV in Sub-Saharan Africa (SSA). This review aims to map the existing literature on SV victimization among university students in SSA, related response strategies, and identify gaps in the evidence.

Methods and Analysis: This review will follow JBI guidelines and will be conducted from 1st July to 31st December 2023. A team of 5 reviewers will screen eligible documents and articles for relevance from various data sources including electronic databases such as MEDLINE, EMBASE, PsychINFO, CINAHL, Google Scholar, PubMed, and websites for government and agencies. Standard information for each study will be collected and a common analytical framework for all the primary documents will be conducted.

Ethics and dissemination: The results will be published in a peer-reviewed journal.

Registration: This review has been registered with the Open Science Framework.

For peer review only - http://bmjopen.bmj.com/site/about/guidelines.xhtml

Keywords: Sexual violence, sexual offenses, response services, university students, Sub-Saharan Africa

Word count: 2140

Article summary

Strengths and limitations of this study

- The review approach will cover information from broad sources, therefore, providing broader synthetic information on SV victimization and response mechanisms on university campuses in sub-Saharan Africa.
- Studies and documents that will be included in this review are conducted and prepared by other people and therefore may include the risk of bias.
- The review will be limited to articles and documents published in the English and French languages only and therefore will lack evidence from documents and articles published in other languages.

INTRODUCTION

Sexual violence (SV) is a serious global public health problem and a violation of sexual rights affecting millions worldwide [1]. SV is defined as any sexual act, attempt to obtain a sexual act, unwanted sexual comments or advances, or acts to traffic, or otherwise directed, against a person's sexuality using coercion, by any person regardless of their relationship to the victim, in any setting, including but not limited to home and work [2]. SV is commonly reported on university campuses around the world with alarmingly high estimates [3]. Globally, the prevalence rate of sexual assault on undergraduates has been estimated at 10.3% in women and 3.1% in men [4]. Studies from SSA indicate that the prevalence of various forms of SV and sexual harassment experienced by university students in five countries vary within and across countries from 14.3% to 78.2% [5-9].

There are several risk factors for SV victimization in university settings. Young women may have sex with a teacher or someone else they are dependent on because they need to pass a course to get financial support, in that way, promotes their subordination and dependency on men [10, 11]. Individual factors like young age, alcohol and drug consumption, year of study, and living out of campus has also being associated with SV victimization.[12] Other factors include previous SV victimization, multiple sex partners, and losing a parent before age two [5, 8, 9, 12, 13]. Weak monitoring systems, lack of guidelines and unprofessional administration, and community attitudes are some of the institutional and community-level factors that are associated with SV victimization [12].

Several initiatives are implemented to address SV in universities; these include development of policies and guidelines to prevent and respond to SV, accessible and trauma-informed services for supporting student disclosure, and advocacy [14]. However, these initiatives meet number of challenges incuding the unintended consequences of mandatory reporting that may include secondary victimization and stigma, and lack of knowledge about available resources on the campus [3] [15].

Despite the reported high rates of SV victimization on campuses in SSA countries,[5-9] the available evidence has not been synthesized to provide an overall picture and a clear understanding of not only the extent of the problem but also knowledge of potential victims, and perpetrators and what

For peer review only - http://bmjopen.bmj.com/site/about/guidelines.xhtml

strategies/interventions have been implemented to respond to SV in SSA universities. Moreover, at the time of writing this protocol, there was no retrieved evidence synthesis or review of existing evidence in form of scoping or systematic review conducted in the area of SV victimization and response among university students across SAA countries from the Joanna Briggs Institute (JBI) Database of Systematic Reviews and Implementation Reports, Cochrane Library or MEDLINE via Ovid website. In response to this gap, this scoping review will conduct a mapping of available evidence on SV victimization on campuses and different strategies used to respond to/address it in SSA universities. In addition, this scoping review will provide preliminary information to guide our research agenda.

Aim, objectives, and research questions

The overall aim of this scoping review is to map existing literature on SV victimization among university students in SSA and related response strategies and to identify gaps in the evidence. This review will provide an evidence basis for specific recommendations in future work and clearly, state gaps in the literature needed to guide the research agenda.

The specific objectives of this scoping review are.

- Map existing literature on SV prevalence, types, risk factors, and common victims and perpetrators of SV among university students on campuses in SSA.
- Describe different laws, policies, strategies, and actions taken to address and prevent SV on campuses and
- Identify evidence gaps.

The main questions guiding this scoping are:

1. What does existing literature tell us about SV among university students in SSA and related responses?
2. What is the gap in knowledge of the existing literature on SV victimization and response among university students in SSA?

METHODS

The review has been registered with the Open Science Framework and will be conducted from 1st July 2023 to 31 December 2023. The review will follow JBI guidelines for scoping reviews [16]. These guidelines were developed from the framework proposed by Arksey and O'Malley's[17] and further enhanced by Levac, Colquhoun, and O'Brien who provided more details of what should be done in each stage of the review process [18]. The JBI guidelines not only propose a detailed and comprehensive step by step process of conducting scoping review but also they are highly recommended to ensure a high quality review in a clear and transparent process [19]. The review will be reported according to the Preferred Reporting Items for Systematic Reviews, and Meta-analyses extension for scoping review (PRISMA-ScR) checklist [20]. With regards to this, the steps towards conducting this review will include 1) search strategy, 2) study selection, 3) charting the data, and 4) collating, 5) summarizing, and 6) reporting the results. The details of what will be done in each step are explained below.

For peer review only - http://bmjopen.bmj.com/site/about/guidelines.xhtml

For peer review only

Information sources and search strategy

The reviewers will utilize the JBI-proposed three-step search strategy to identify relevant articles for this study [16]. The reviewers will first start by searching from two databases (MEDLINE and CINAHL) using the title of this review. The reviewers will then analyze the text words contained in the title and abstract of retrieved papers, and of the index, terms used to describe the articles. The second step will focus on all index terms and identified keywords from the titles and abstracts of the retrieved papers. These index terms will then be used to search all selected information sources including electronic databases such as MEDLINE, EMBASE, PsychINFO, CINAHL, Google Scholar, and PubMed. Additional sources, search terms, and keywords may be discovered and incorporated into the search strategy. The reviewers will also search for other sources and other documents including government policies and guidelines from government websites and local and international agencies that will be identified during the search process. In the third step, reviewers will use the reference list of all selected articles to search for additional studies and include them in the list. The list of all identified studies and documents will be uploaded to the EndNote reference manager software version 20.5 (clarivate), and duplicates will be removed. To minimize reporting bias, this scoping review will be conducted by two independent primary reviewers and two secondary reviewers. Reviewers will contact authors of primary studies, reviews, and policy documents for further information when additional information is required.

Eligibility criteria

As proposed by the JBI guidelines,[16] we used the population, concepts, and context (PCC) approach to formulate our inclusion criteria for the studies that will be reviewed.

Inclusion criteria

Population: The target population for this review will be the human subjects who are university students, both males and females, aged 18 years and above.

Concept: Our core concept for this review is SV and we will focus on its/in relation to prevalence, types, risk factors, common victims, and perpetrators as well as laws, policies, strategies, and actions taken to address SV on campuses.

Context: This review will be limited to studies conducted in universities in SSA and policy documents addressing sexual violence in Universities in SSA, from the inception until 30th November 2023.

Exclusion criteria: Studies conducted in settings other than Univerities, that focused on areas other than SSA, populations other than studentsnot focusing on SV in relation to the concepts listed above and those published in another language other than English or French will be excluded.

Study selection

Reviewers will assess the retrieved articles for relevance using the inclusion criteria. This will be done in two steps, first by screening the title and abstracts and then by full text whereby copies of full articles will be retrieved. A list of those studies that will meet the inclusion criteria will be selected. At this stage, reviewers will convene and jointly discuss any discrepancies between the two lists. Secondary reviewers will be invited and consulted to resolve any discrepancies before moving to the next stage. The reviewers will then read the full article for assessment against the inclusion criteria for the review. The detailed flow chart of all studies that were included and excluded, with clear reasons for exclusion will be developed and included in our report. Any disagreements that arise between the reviewers at this stage of the selection process will be resolved through discussion with secondary reviewers.

For peer review only - http://bmjopen.bmj.com/site/about/guidelines.xhtml

Charting the data

Reviewers will start by reading the primary research articles and policy documents and collecting standard information about each article. Reviewers will then extract information and enter it in the prepared data charting form using the database program Excel. Information that will be initially entered in the data charting form will include the author(s), year of publication, study location (country), study population, aims of the study, methodology, data collection tools, outcome measures, and key findings. Thereafter, the reviewers will make a broader narrative review to analyze information based on the context of the study findings and their conclusion. The charts will contain both numerical summaries and qualitative thematic analysis findings.

Collating, summarizing, and reporting the results.

In this stage, reviewers will focus on identifying the implications of the study findings for policy, practice, or research. All articles included in the review will be screened for results. We will develop a thematic framework that will enable us to present a narrative account of all extracted information from the articles based on overlaps and diversities of the findings to answer our research questions.

Summarizing

Depending on the findings from the studies we will select the best way to organize and summarize that data, these can include figures, tables, and evidence gaps maps, in a diagrammatic or tabular form, and/or in a descriptive format that aligns with our objectives and elements of the PCC as mentioned above. Descriptive analysis of the extracted data will be done, the type of descriptive analysis will depend more on the type of extracted data.

Reporting the results

The decision of which method of reporting to be used will be done by involving all reviewers in the team. The outcome of this review is expected to include findings on SV victimization among university students in SSA countries and related information that will be mapped in our findings like characteristics of victims and perpetrators. Another outcome will be response systems following SV victimization and the characteristics of the response system, the structures and users, and the strength and gaps. The policy documents and guidelines will also be reviewed, and details of implementations that have been done, the strengths, as well as the gaps, will be summarized in this review.

ETHICS AND DISSEMINATION

We anticipate that these results will be disseminated through publication in a peer-reviewed journal and conferences targeting researchers, programmers, policymakers, responders, and university students across the continent. The dataset will be made available at the Umea University and the Muhimbili University of Health and Allied Science repositories. We expect that the findings of this scoping review will provide important information on SV prevalence, types, risk factors, common victims and perpetrators, laws, policies, strategies, and actions taken to address SV on campuses in SSA that will guide future work. The findings will further delimit the gaps in the published data to guide the forthcoming research agenda in SSA, particularly in Tanzania.

Patient and Public Involvement

None

For peer review only

ACKNOWLEDGMENTS

Funding statement

This work was supported by the Erling Persson's Foundation (EP) through the Department of Epidemiology and Global Health (EpiGH), Umeå University for training and the Muhimbili University of Health and Allied Sciences by providing a protected time for the corresponding author to conduct this review.

Author contributions

ESM conceptualized the review approach and wrote the first draft of the manuscript. RCM reviewed and provided inputs in the methods section. FNKN identified the framework and provided general guidance in all sections. ML and SK reviewed the framework and provided inputs on the scope of the review and methods. All authors read and approved the final manuscript.

Authors' information

ESM is a doctoral candidate at the Department of Epidemiology and Global Health, Umea University, SWEDEN, and a Psychiatrist at the Department of Psychiatry and Mental Health, Muhimbili University of Health and Allied Sciences
Email: ester.mzilangwe@umu.se, ester.steven@muhas.ac.tz
Address: 9 United Nations Road; Upanga West; P.O. Box 65001, Dar es Salaam, TANZANIA

RCM is a Senior Librarian at the Directorate of Library Services, Muhimbili University of Health and Allied Sciences
Email: rchandemallya@muhas.ac.tz, rchandemallya@gmail.com

ML is an Associate Professor and Director of Studies at the Department of Epidemiology and Global Health, Umea University, SWEDEN
Email: marie.lindkvist@umu.se

SK is a Professor of Psychiatry at the Department of Psychiatry and Mental Health, Muhimbili University of Health and Allied Sciences
Email: skaaya@gmail.com

FNKN is a Senior Research Associate at the Department of Epidemiology and Global Health, Umea University, SWEDEN
Email: faustine.nkulu@umu.se

Competing Interests Statement

The authors declare no competing interest.

For peer review only

REFERENCES

1. WHO, *Global status report on violence prevention 2014*. 2014: World Health Organization.
2. Krug, E.G., et al., *The world report on violence and health.* The Lancet, 2002. **360**(9339): p. 1083-1088.
3. Halstead, V., J.R. Williams, and R. Gonzalez-Guarda, *Sexual violence in the college population: a systematic review of disclosure and campus resources and services.* J Clin Nurs, 2017. **26**(15-16): p. 2137-2153.
4. Dills J, F.D., Payne G., *Sexual Violence on Campus: Strategies for Prevention. .* Atlanta, GA: National Center for Injury Prevention and Control, Centers for Disease Control and Prevention, 2016.
5. Mezie-Okoye, M.-M.I. and F. Alamina, *Sexual violence among female undergraduates in a tertiary institution in Port Harcourt: prevalence, pattern, determinants and health consequences.* African journal of reproductive health, 2014. **18**(4): p. 79-85.
6. Ogunbode, O.O., F.A. Bello, and A.M. Ogunbode, *Sexual violence against female undergraduates in a Nigerian tertiary institution.* Tropical Journal of Obstetrics and Gynaecology, 2014. **31**(1): p. 99-109.
7. Agardh, A., E. Cantor-Graae, and P.O. Ostergren, *Youth, sexual risk-taking behavior, and mental health: a study of university students in Uganda.* Int J Behav Med, 2012. **19**(2): p. 208-16.
8. Fielding-Miller, R., et al., *Epidemiology of campus sexual assault among university women in Eswatini.* Journal of interpersonal violence, 2019: p. 0886260519888208.
9. Norman, I.D., M. Aikins, and F.N. Binka, *Sexual harassment in public medical schools in Ghana.* Ghana Medical Journal, 2013. **47**(3): p. 128-136.
10. Kaufman, C.E. and S.E. Stavrou, *'Bus fare please': the economics of sex and gifts among young people in urban South Africa.* Culture, Health & Sexuality, 2004. **6**(5): p. 377-391.
11. Zembe, Y.Z., et al., *"Money talks, bullshit walks" interrogating notions of consumption and survival sex among young women engaging in transactional sex in post-apartheid South Africa: a qualitative enquiry.* Globalization and Health, 2013. **9**(1): p. 28.
12. Dranzoa, C., *Sexual Harassment at African Higher Education Institutions.* International Higher Education, 2018. **94**: p. 4.
13. Temesgan, W.Z., Z.M. Endale, and G.L. Aynalem, *Sexual violence and associated factors among female night college students after joining the college in Debre Markos town, North West Ethiopia, 2019.* Clinical Epidemiology and Global Health, 2021. **10**.
14. *Tertiary Education Quality and Standards Agency, Good Practice Note: Preventing and responding to sexual assault and sexual harassment in the Australian higher education sector.* July 2020.
15. Warner, W.P.J., *Sexual Violence Response and Prevention Studies of Campus Policies and Practices.* Journal of School Violence, 2017.
16. Peters, M.D., et al., *The Joanna Briggs Institute reviewers' manual 2015: methodology for JBI scoping reviews.* 2015.
17. Arksey, H. and L. O'Malley, *Scoping studies: towards a methodological framework.* International Journal of Social Research Methodology, 2005. **8**(1): p. 19-32.
18. Levac, D., H. Colquhoun, and K.K. O'Brien, *Scoping studies: advancing the methodology.* Implementation science, 2010. **5**: p. 1-9.
19. Khalil, H., et al., *Conducting high quality scoping reviews-challenges and solutions.* Journal of clinical epidemiology, 2021. **130**: p. 156-160.

For peer review only - http://bmjopen.bmj.com/site/about/guidelines.xhtml

20. Tricco, A.C., et al., *PRISMA extension for scoping reviews (PRISMA-ScR): checklist and explanation.* Annals of internal medicine, 2018. **169**(7): p. 467-473.

For peer review only

For peer review only - http://bmjopen.bmj.com/site/about/guidelines.xhtml

Sexual violence victimization and response among university students in sub-Saharan Africa: A scoping review protocol

Journal:	*BMJ Open*
Manuscript ID	bmjopen-2023-076015.R2
Article Type:	Protocol
Date Submitted by the Author:	03-Jan-2024
Complete List of Authors:	Mzilangwe, Ester; Umea Universitet, Department of Epidemiology and Global Health; Muhimbili University of Health and Allied Sciences, Psychiatry and Mental Health<br>Chande Mallya, Rehema; Muhimbili University of Health and Allied Sciences, Directorate of Library Services<br>Lindkvist, Marie; Umea Universitet, Department of Epidemiology and Global Health<br>Kaaya, Sylvia; Muhimbili University of Health and Allied Sciences, Department of Psychiatry and Mental Health<br>Nkulu Kalengayi, Faustine Kyungu; Umea Universitet, Epidemiology and Global Health
**Primary Subject Heading**:	Public health
Secondary Subject Heading:	Health policy, Sexual health
Keywords:	Health Services Accessibility, Health Equity, PUBLIC HEALTH, Health policy < HEALTH SERVICES ADMINISTRATION & MANAGEMENT, Organisation of health services < HEALTH SERVICES ADMINISTRATION & MANAGEMENT, Risk Factors

Sexual violence victimization and response among university students in sub-Saharan Africa: A scoping review protocol

Ester Steven Mzilangwe[1,2], Rehema Chande[1], Marie Lindkvist[2], Sylvia Kaaya[1], Faustine Kyungu Nkulu Kalengayi[2]

1. Department of Psychiatry and Mental Health and Directorate of Library Services, Muhimbili University of Health and Allied Sciences, Tanzania
2. Department of Epidemiology and Global Health, Umea University, Sweden

Corresponding Author: Ester Steven Mzilangwe,
Email: ester.mzilangwe@umu.se, ester.steven@muhas.ac.tz
Telephone number: +255788202020
Address: 9 United Nations Road; Upanga West; P.O. Box 65001, Dar es Salaam, TANZANIA.

Data statement: The dataset of this review will be made available at the Umea University and the Muhimbili University of Health and Allied Science repositories http://dspace.muhas.ac.tz:8080/xmlui/

ABSTRACT

Introduction: Sexual violence (SV) is highly prevalent among university campuses across the globe, resulting in devastating educational and health consequences. Several studies have been published focusing on various aspects of SV on campuses. However, no review have been retrieved from the Joanna Briggs Institute (JBI) Database, Cochrane Library, or Ovid examining evidence synthesis on prevalence, risk factors, victims and perpetrators, policies, laws, and universities responses to SV in Sub-Saharan Africa (SSA). This review aims to map the existing literature on SV victimization among university students in SSA, related response strategies, and identify gaps in the evidence.

Methods and Analysis: This review will follow JBI guidelines and will be conducted from 1st July to 31st December 2023. A team of five reviewers will screen eligible documents and articles for relevance from various data sources including electronic databases such as MEDLINE, EMBASE, PsychINFO, CINAHL, Google Scholar, PubMed, and websites for government and agencies. Standard information for each study will be collected and a common analytical framework for all the primary documents will be conducted.

Ethics and dissemination: This review will involve analysis of published data only and therefore does not require ethics approval. The results will be published in a peer-reviewed journal.

Registration: This review has been registered with the Open Science Framework.

For peer review only - http://bmjopen.bmj.com/site/about/guidelines.xhtml

Keywords: Sexual violence, sexual offenses, response services, university students, Sub-Saharan Africa

Word count: 2189

Article summary

Strengths and limitations of this study

- The review approach will cover information from broad sources, therefore, providing broader synthetic information on SV victimization and response mechanisms on university campuses in sub-Saharan Africa.
- Studies and documents that will be included in this review are conducted and prepared by other people and therefore may include the risk of bias.
- The review will be limited to articles and documents published in the English and French languages only and therefore will lack evidence from documents and articles published in other languages.

INTRODUCTION

Sexual violence (SV) is a serious global public health problem and a violation of sexual rights affecting millions worldwide [1]. SV is defined as any sexual act, attempt to obtain a sexual act, unwanted sexual comments or advances, or acts to traffic, or otherwise directed, against a person's sexuality using coercion, by any person regardless of their relationship to the victim, in any setting, including but not limited to home and work [2]. SV is commonly reported on university campuses around the world with alarmingly high estimates [3]. Globally, the prevalence rate of sexual assault on undergraduates has been estimated at 10.3% in women and 3.1% in men [4]. Studies from SSA indicate that the prevalence of various forms of SV and sexual harassment experienced by university students in five countries vary within and across countries from 14.3% to 78.2% [5-9].
There are several risk factors for SV victimization in university settings. Young women may have sex with a teacher or someone else they are dependent on because they need to pass a course to get financial support, in that way, promotes their subordination and dependency on men [10, 11]. Individual factors like young age, alcohol and drug consumption, year of study, and living out of campus has also being associated with SV victimization.[12] Other factors include previous SV victimization, multiple sex partners, and losing a parent before age two [5, 8, 9, 12, 13]. Weak monitoring systems, lack of guidelines and unprofessional administration, and community attitudes are some of the institutional and community-level factors that are associated with SV victimization [12].
Several initiatives are implemented to address SV in universities; these include development of policies and guidelines to prevent and respond to SV, accessible and trauma-informed services for supporting student disclosure, and advocacy [14]. However, these initiatives meet number of challenges incuding the unintended consequences of mandatory reporting that may include secondary victimization and stigma, and lack of knowledge about available resources on the campus [3] [15].

Despite the reported high rates of SV victimization on campuses in SSA countries,[5-9] the available evidence has not been synthesized to provide an overall picture and a clear understanding of not only the extent of the problem but also knowledge of potential victims, and perpetrators and what

For peer review only - http://bmjopen.bmj.com/site/about/guidelines.xhtml

strategies/interventions have been implemented to respond to SV in SSA universities. Moreover, at the time of writing this protocol, there was no retrieved evidence synthesis or review of existing evidence in form of scoping or systematic review conducted in the area of SV victimization and response among university students across SAA countries from the Joanna Briggs Institute (JBI) Database of Systematic Reviews and Implementation Reports, Cochrane Library or MEDLINE via Ovid website. In response to this gap, this scoping review will conduct a mapping of available evidence on SV victimization on campuses and different strategies used to respond to/address it in SSA universities. In addition, this scoping review will provide preliminary information to guide our research agenda.

Aim, objectives, and research questions

The overall aim of this scoping review is to map existing literature on SV victimization among university students in SSA and related response strategies and to identify gaps in the evidence. This review will provide an evidence basis for specific recommendations in future work and clearly, state gaps in the literature needed to guide the research agenda.

The specific objectives of this scoping review are.

- Map existing literature on SV prevalence, types, risk factors, and common victims and perpetrators of SV among university students on campuses in SSA.
- Describe different laws, policies, strategies, and actions taken to address and prevent SV on campuses and
- Identify evidence gaps.

The main questions guiding this scoping are:

1. What does existing literature tell us about SV prevalence, types, risk factors, and common victims and perpetrators of SV on campuses in SSA?
2. What are the various laws, policies, strategies, and actions that have been implemented to address and prevent SV against university students on campuses in SSA?
3. What is the gap in knowledge of the existing literature on SV victimization and response among university students on campuses in SSA?

METHODS

The review has been registered with the Open Science Framework and will be conducted from 1st July 2023 to 31 December 2023. The review will follow JBI guidelines for scoping reviews [16]. These guidelines were developed from the framework proposed by Arksey and O'Malley's[17] and further enhanced by Levac, Colquhoun, and O'Brien who provided more details of what should be done in each stage of the review process [18]. The JBI guidelines not only propose a detailed and comprehensive step by step process of conducting scoping review but also they are highly recommended to ensure a high quality review in a clear and transparent process [19]. The review will be reported according to the Preferred Reporting Items for Systematic Reviews, and Meta-analyses extension for scoping review (PRISMA-ScR) checklist [20]. With regards to this, the steps towards conducting this review will include 1) search strategy, 2) study selection, 3) charting the data, and 4) collating, 5) summarizing, and 6) reporting the results. The details of what will be done in each step are explained below.

For peer review only - http://bmjopen.bmj.com/site/about/guidelines.xhtml

Information sources and search strategy

The reviewers will utilize the JBI-proposed three-step search strategy to identify relevant articles for this study [16]. The reviewers will first start by searching from two databases (MEDLINE and CINAHL) using the title of this review. The reviewers will then analyze the text words contained in the title and abstract of retrieved papers, and of the index, terms used to describe the articles. The second step will focus on all index terms and identified keywords from the titles and abstracts of the retrieved papers. These index terms will then be used to search all selected information sources including electronic databases such as MEDLINE, EMBASE, PsychINFO, CINAHL, Google Scholar, and PubMed. The complete electronic search strategy for all database can be found under supplementary materials, further search strategy details across bibliographic databases are available upon request from the first author. Additional sources, search terms, and keywords may be discovered and incorporated into the search strategy. The reviewers will also search for other sources and other documents including government policies and guidelines from government websites and local and international agencies that will be identified during the search process. In the third step, reviewers will use the reference list of all selected articles to search for additional studies and include them in the list. The list of all identified studies and documents will be uploaded to the EndNote reference manager software version 20.5 (clarivate), and duplicates will be removed. To minimize reporting bias, this scoping review will be conducted by two independent primary reviewers and two secondary reviewers. Reviewers will contact authors of primary studies, reviews, and policy documents for further information when additional information is required.

Eligibility criteria

As proposed by the JBI guidelines,[16] we used the population, concepts, and context (PCC) approach to formulate our inclusion criteria for the studies that will be reviewed.

Inclusion criteria

Population: The target population for this review will be the human subjects who are university students, both males and females, aged 18 years and above.

Concept: Our core concept for this review is SV and we will focus on its/in relation to prevalence, types, risk factors, common victims, and perpetrators as well as laws, policies, strategies, and actions taken to address SV on campuses.

Context: This review will be limited to studies conducted in universities in SSA and policy documents addressing sexual violence in Universities in SSA, from the inception until 31st December 2023.

Exclusion criteria: Studies conducted in settings other than Universities, that focused on areas other than SSA, populations other than students not focusing on SV in relation to the concepts listed above and those published in another language other than English or French will be excluded.

Study selection

Reviewers will assess the retrieved articles for relevance using the inclusion criteria. This will be done in two steps, first by screening the title and abstracts and then by full text whereby copies of full articles will be retrieved. A list of those studies that will meet the inclusion criteria will be selected. At this stage, reviewers will convene and jointly discuss any discrepancies between the two lists. Secondary reviewers will be invited and consulted to resolve any discrepancies before moving to the next stage. The reviewers will then read the full article for assessment against the inclusion criteria for the review. The detailed flow chart of all studies that were included and excluded, with clear reasons for exclusion

For peer review only - http://bmjopen.bmj.com/site/about/guidelines.xhtml

will be developed and included in our report. Any disagreements that arise between the reviewers at this stage of the selection process will be resolved through discussion with secondary reviewers.

Charting the data

Reviewers will start by reading the primary research articles and policy documents and collecting standard information about each article. Reviewers will then extract information and enter it in the prepared data charting form using the database program Excel. Information that will be initially entered in the data charting form will include the author(s), year of publication, study location (country), study population, aims of the study, methodology, data collection tools, outcome measures, and key findings. Thereafter, the reviewers will make a broader narrative review to analyze information based on the context of the study findings and their conclusion. The charts will contain both numerical summaries and qualitative thematic analysis findings.

Collating, summarizing, and reporting the results.

In this stage, reviewers will focus on identifying the implications of the study findings for policy, practice, or research. All articles included in the review will be screened for results. We will develop a thematic framework that will enable us to present a narrative account of all extracted information from the articles based on overlaps and diversities of the findings to answer our research questions.

Summarizing

Depending on the findings from the studies we will select the best way to organize and summarize that data, these can include figures, tables, and evidence gaps maps, in a diagrammatic or tabular form, and/or in a descriptive format that aligns with our objectives and elements of the PCC as mentioned above. Descriptive analysis of the extracted data will be done, the type of descriptive analysis will depend more on the type of extracted data.

Reporting the results

The decision of which method of reporting to be used will be done by involving all reviewers in the team. The outcome of this review is expected to include findings on SV victimization among university students in SSA countries and related information that will be mapped in our findings like characteristics of victims and perpetrators. Another outcome will be response systems following SV victimization and the characteristics of the response system, the structures and users, and the strength and gaps. The policy documents and guidelines will also be reviewed, and details of implementations that have been done, the strengths, as well as the gaps, will be summarized in this review.

ETHICS AND DISSEMINATION

Since this review will only involve analysis of published data, it does not require ethics approval. We anticipate that these results will be disseminated through publication in a peer-reviewed journal and conferences targeting researchers, programmers, policymakers, responders, and university students across the continent. The dataset will be made available at the Umea University and the Muhimbili University of Health and Allied Science repositories. We expect that the findings of this scoping review will provide important information on SV prevalence, types, risk factors, common victims and perpetrators, laws, policies, strategies, and actions taken to address SV on campuses in SSA that will guide future work. The findings will further delimit the gaps in the published data to guide the forthcoming research agenda in SSA.

For peer review only - http://bmjopen.bmj.com/site/about/guidelines.xhtml

For peer review only

Patient and Public Involvement

None

ACKNOWLEDGMENTS

Funding statement
This work was supported by the Erling Persson's Foundation (EP) through the Department of Epidemiology and Global Health (EpiGH), Umeå University for training and the Muhimbili University of Health and Allied Sciences by providing a protected time for the corresponding author to conduct this review.

Author contributions
ESM conceptualized the review approach and wrote the first draft of the manuscript. RCM reviewed and provided inputs in the methods section. FNKN identified the framework and provided general guidance in all sections. ML and SK reviewed the framework and provided inputs on the scope of the review and methods. All authors read and approved the final manuscript.

Authors' information
ESM is a doctoral candidate at the Department of Epidemiology and Global Health, Umea University, SWEDEN, and a Psychiatrist at the Department of Psychiatry and Mental Health, Muhimbili University of Health and Allied Sciences
Email: ester.mzilangwe@umu.se, ester.steven@muhas.ac.tz
Address: 9 United Nations Road; Upanga West; P.O. Box 65001, Dar es Salaam, TANZANIA

RCM is a Senior Librarian at the Directorate of Library Services, Muhimbili University of Health and Allied Sciences
Email: rchandemallya@muhas.ac.tz, rchandemallya@gmail.com

ML is an Associate Professor and Director of Studies at the Department of Epidemiology and Global Health, Umea University, SWEDEN
Email: marie.lindkvist@umu.se

SK is a Professor of Psychiatry at the Department of Psychiatry and Mental Health, Muhimbili University of Health and Allied Sciences
Email: skaaya@gmail.com

FNKN is a Senior Research Associate at the Department of Epidemiology and Global Health, Umea University, SWEDEN
Email: faustine.nkulu@umu.se

Competing Interests Statement

The authors declare no competing interest.

For peer review only

REFERENCES

1. WHO, *Global status report on violence prevention 2014*. 2014: World Health Organization.
2. Krug, E.G., et al., *The world report on violence and health.* The Lancet, 2002. **360**(9339): p. 1083-1088.
3. Halstead, V., J.R. Williams, and R. Gonzalez-Guarda, *Sexual violence in the college population: a systematic review of disclosure and campus resources and services.* J Clin Nurs, 2017. **26**(15-16): p. 2137-2153.
4. Dills J, F.D., Payne G., *Sexual Violence on Campus: Strategies for Prevention. .* Atlanta, GA: National Center for Injury Prevention and Control, Centers for Disease Control and Prevention, 2016.
5. Mezie-Okoye, M.-M.I. and F. Alamina, *Sexual violence among female undergraduates in a tertiary institution in Port Harcourt: prevalence, pattern, determinants and health consequences.* African journal of reproductive health, 2014. **18**(4): p. 79-85.
6. Ogunbode, O.O., F.A. Bello, and A.M. Ogunbode, *Sexual violence against female undergraduates in a Nigerian tertiary institution.* Tropical Journal of Obstetrics and Gynaecology, 2014. **31**(1): p. 99-109.
7. Agardh, A., E. Cantor-Graae, and P.O. Ostergren, *Youth, sexual risk-taking behavior, and mental health: a study of university students in Uganda.* Int J Behav Med, 2012. **19**(2): p. 208-16.
8. Fielding-Miller, R., et al., *Epidemiology of campus sexual assault among university women in Eswatini.* Journal of interpersonal violence, 2019: p. 0886260519888208.
9. Norman, I.D., M. Aikins, and F.N. Binka, *Sexual harassment in public medical schools in Ghana.* Ghana Medical Journal, 2013. **47**(3): p. 128-136.
10. Kaufman, C.E. and S.E. Stavrou, *'Bus fare please': the economics of sex and gifts among young people in urban South Africa.* Culture, Health & Sexuality, 2004. **6**(5): p. 377-391.
11. Zembe, Y.Z., et al., *"Money talks, bullshit walks" interrogating notions of consumption and survival sex among young women engaging in transactional sex in post-apartheid South Africa: a qualitative enquiry.* Globalization and Health, 2013. **9**(1): p. 28.
12. Dranzoa, C., *Sexual Harassment at African Higher Education Institutions.* International Higher Education, 2018. **94**: p. 4.
13. Temesgan, W.Z., Z.M. Endale, and G.L. Aynalem, *Sexual violence and associated factors among female night college students after joining the college in Debre Markos town, North West Ethiopia, 2019.* Clinical Epidemiology and Global Health, 2021. **10**.
14. *Tertiary Education Quality and Standards Agency, Good Practice Note: Preventing and responding to sexual assault and sexual harassment in the Australian higher education sector.* July 2020.
15. Warner, W.P.J., *Sexual Violence Response and Prevention Studies of Campus Policies and Practices.* Journal of School Violence, 2017.
16. Peters, M.D., et al., *The Joanna Briggs Institute reviewers' manual 2015: methodology for JBI scoping reviews.* 2015.
17. Arksey, H. and L. O'Malley, *Scoping studies: towards a methodological framework.* International Journal of Social Research Methodology, 2005. **8**(1): p. 19-32.
18. Levac, D., H. Colquhoun, and K.K. O'Brien, *Scoping studies: advancing the methodology.* Implementation science, 2010. **5**: p. 1-9.
19. Khalil, H., et al., *Conducting high quality scoping reviews-challenges and solutions.* Journal of clinical epidemiology, 2021. **130**: p. 156-160.

For peer review only - http://bmjopen.bmj.com/site/about/guidelines.xhtml

20. Tricco, A.C., et al., *PRISMA extension for scoping reviews (PRISMA-ScR): checklist and explanation.* Annals of internal medicine, 2018. **169**(7): p. 467-473.

For peer review only

For peer review only - http://bmjopen.bmj.com/site/about/guidelines.xhtml

Electronic search strategy

Our initial search involved two databases (MEDLINE and CINAHL) using the title of this review. We then analyze the text words contained in the title and abstract of retrieved papers, and of the index, terms used to describe the articles. Here we present **full electronic initial search strategy** for MEDLINE and CINAHL database.

Full electronic search strategy for MEDLINE database.

Database [platform]	Search terms	Limiters/Expanders
Medline [EBSCO]	(((MM "Sex Offenses+") OR (MH "Violence+") OR (MH "Sexual Trauma") OR (MM "Sexual Harassment") OR "sexual violence" OR (MH "Gender-Based Violence") OR "sexual abuse" OR "sexual assault" OR rape)) AND (((MM "Universities") OR (MM "Students") OR "university students" OR (MH "Students, Dental") OR (MH "Students, Public Health") OR (MH "Students, Health Occupations") OR (students in higher education) OR undergraduate OR campus))	Expanders - Apply related words; Apply equivalent subjects<br>Narrow by SubjectGeographic: - africa<br>Narrow by SubjectAge: - adult: 19-44 years<br>Narrow by Language: - english<br>Search modes - Find all my search terms
CINHAL [EBSCO]	(((MM "Sex Offenses+") OR (MH "Violence+") OR (MH "Sexual Trauma") OR (MM "Sexual Harassment") OR "sexual violence" OR (MH "Gender-Based Violence") OR "sexual abuse" OR "sexual assault" OR rape)) AND (((MM "Universities") OR (MM "Students") OR "university students" OR (MH "Students, Dental") OR (MH "Students, Public Health") OR (MH "Students, Health Occupations") OR (students in higher education) OR undergraduate OR campus))	Expanders - Apply related words; Apply equivalent subjects<br>Narrow by SubjectGeographic: - africa<br>Narrow by SubjectAge: - adult: 19-44 years<br>Narrow by Language: - english<br>Search modes - Find all my search terms
ACADEMIC SEARCH PREMIER	(((MM "Sex Offenses+") OR (MH "Violence+") OR (MH "Sexual Trauma") OR (MM "Sexual Harassment") OR "sexual violence" OR (MH "Gender-Based Violence") OR "sexual abuse" OR "sexual assault" OR rape)) AND (((MM "Universities") OR (MM "Students")	Expanders - Apply related words; Apply equivalent subjects<br>Search modes - Find all my search terms<br>Narrow by Language: - english<br>Search modes - Find all my search terms

For peer review only

	OR "university students" OR (MH "Students, Dental") OR (MH "Students, Public Health") OR (MH "Students, Health Occupations") OR (students in higher education) OR undergraduate OR campus))	Expanders - Apply related words; Apply equivalent subjects<br>Narrow by SubjectGeographic: - africa
EMBASE [EBSCO]	#1. 'gender violence' AND 'higher learning institution'<br>#2. ('gender violence' OR (('gender'/exp OR gender) AND ('violence'/exp OR violence))) AND ('higher learning institution' OR (higher AND ('learning'/exp OR learning) AND institution))<br>#3. (sexual AND ('violence'/exp OR violence) OR 'sexual harassment'/exp OR 'sexual harassment' OR (('sex'/exp OR sex) AND offence) OR (('gender'/exp OR gender) AND based AND ('violence'/exp OR violence)) OR (sexual AND ('abuse'/exp OR abuse)) OR (sexual AND ('assault'/exp OR assault)) OR 'rape'/exp OR rape) AND (university:ti AND students:ti OR undergraduate:ti OR (college:ti AND student:ti) OR (students:ti AND in:ti AND higher:ti AND education:ti))	#4. #3 AND 'human'/de<br>#5. #4 AND [adult]/lim
PubMed [EBSCO]	(("sexual violence"[Title] OR "sexual harassment"[Title] OR "sex offence*"[Title] OR "gender based violence"[Title] OR "sexual abuse"[Title] OR "sexual assault"[Title] OR "rape"[Title]) AND ("university student*"[Title] OR "undergraduate*"[Title] OR "college student*"[Title] OR ("student*"[All Fields] AND "in higher education"[Title]))) AND ((humans[Filter]) AND (english[Filter]) AND (adult[Filter]))	Humans, English, Adult: 19-44 years
Google	What are African governments doing about sexual violence on campus?	Websites/examples:<br>https://www.globalcitizen.org/es/content/south-africa-university-gender-violence-pandor/

For peer review only

		https://www.un.org/africarenewal/magazine/august-november-2018/confronting-sexual-violence-schools<br>https://aau.org/ (Association of African Universities)<br>https://essa-africa.org/ (Education Sub Saharan Africa)

For peer review only

Preferred Reporting Items for Systematic reviews and Meta-Analyses extension for Scoping Reviews (PRISMA-ScR) Checklist

SECTION	ITEM	PRISMA-ScR CHECKLIST ITEM	REPORTED ON PAGE #
**TITLE**			
Title	1	Identify the report as a scoping review.	1: line 3 - 6
**ABSTRACT**			
Structured summary	2	Provide a structured summary that includes (as applicable): background, objectives, eligibility criteria, sources of evidence, charting methods, results, and conclusions that relate to the review questions and objectives.	1: line 27-50
**INTRODUCTION**			
Rationale	3	Describe the rationale for the review in the context of what is already known. Explain why the review questions/objectives lend themselves to a scoping review approach.	2: line 29-60<br>3: line 3-12
Objectives	4	Provide an explicit statement of the questions and objectives being addressed with reference to their key elements (e.g., population or participants, concepts, and context) or other relevant key elements used to conceptualize the review questions and/or objectives.	3: line 15-39
**METHODS**			
Protocol and registration	5	Indicate whether a review protocol exists; state if and where it can be accessed (e.g., a Web address); and if available, provide registration information, including the registration number.	3: line 43
Eligibility criteria	6	Specify characteristics of the sources of evidence used as eligibility criteria (e.g., years considered, language, and publication status), and provide a rationale.	4: line 27 - 45
Information sources*	7	Describe all information sources in the search (e.g., databases with dates of coverage and contact with authors to identify additional sources), as well as the date the most recent search was executed.	4: line 6-7, 11-14
Search	8	Present the full electronic search strategy for at least 1 database, including any limits used, such that it could be repeated.	4: line 5-24
Selection of sources of evidence†	9	State the process for selecting sources of evidence (i.e., screening and eligibility) included in the scoping review.	4: line 50 - 60
Data charting process‡	10	Describe the methods of charting data from the included sources of evidence (e.g., calibrated forms or forms that have been tested by the team before their use, and whether data charting was done independently or in duplicate) and any processes for obtaining and confirming data from investigators.	5: line 5 - 14
Data items	11	List and define all variables for which data were sought and any assumptions and simplifications made.	N/A
Critical appraisal of individual sources of evidence§	12	If done, provide a rationale for conducting a critical appraisal of included sources of evidence; describe the methods used and how this information was used in any data synthesis (if appropriate).	N/A
Synthesis of results	13	Describe the methods of handling and summarizing the data that were charted.	5: line 17 - 41

For peer review only - http://bmjopen.bmj.com/site/about/guidelines.xhtml

SECTION	ITEM	PRISMA-ScR CHECKLIST ITEM	REPORTED ON PAGE #
**RESULTS**			
Selection of sources of evidence	14	Give numbers of sources of evidence screened, assessed for eligibility, and included in the review, with reasons for exclusions at each stage, ideally using a flow diagram.	N/A
Characteristics of sources of evidence	15	For each source of evidence, present characteristics for which data were charted and provide the citations.	N/A
Critical appraisal within sources of evidence	16	If done, present data on critical appraisal of included sources of evidence (see item 12).	N/A
Results of individual sources of evidence	17	For each included source of evidence, present the relevant data that were charted that relate to the review questions and objectives.	N/A
Synthesis of results	18	Summarize and/or present the charting results as they relate to the review questions and objectives.	N/A
**DISCUSSION**			
Summary of evidence	19	Summarize the main results (including an overview of concepts, themes, and types of evidence available), link to the review questions and objectives, and consider the relevance to key groups.	N/A
Limitations	20	Discuss the limitations of the scoping review process.	2: line 13 - 23
Conclusions	21	Provide a general interpretation of the results with respect to the review questions and objectives, as well as potential implications and/or next steps.	N/A
**FUNDING**			
Funding	22	Describe sources of funding for the included sources of evidence, as well as sources of funding for the scoping review. Describe the role of the funders of the scoping review.	6: line 5 - 10

Reference

Shamseer L, Moher D, Clarke M, Ghersi D, Liberati A, Petticrew M, Shekelle P, Stewart LA. Preferred reporting items for systematic review and meta-analysis protocols (PRISMA-P) 2015: elaboration and explanation. Bmj. 2015 Jan 2;349.

For peer review only
